# Ethics of selective restriction of liberty in a pandemic

**DOI:** 10.1136/medethics-2020-107104

**Published:** 2021-05-31

**Authors:** James Cameron, Bridget Williams, Romain Ragonnet, Ben Marais, James Trauer, Julian Savulescu

**Affiliations:** 1 Murdoch Childrens Research Institute, Parkville, Victoria, Australia; 2 School of Public Health and Preventative Medicine, Monash University, Clayton, Victoria, Australia; 3 Marie Bashir Institute for Infectious Diseases and Biosecurity, The University of Sydney, Sydney, New South Wales, Australia; 4 Faculty of Philosophy, University of Oxford, Oxford, UK

**Keywords:** COVID-19, ethics, public health ethics

## Abstract

Liberty-restricting measures have been implemented for centuries to limit the spread of infectious diseases. This article considers if and when it may be ethically acceptable to impose selective liberty-restricting measures in order to reduce the negative impacts of a pandemic by preventing particularly vulnerable groups of the community from contracting the disease. We argue that the commonly accepted explanation—that liberty restrictions may be justified to prevent harm to others when this is the least restrictive option—fails to adequately accommodate the complexity of the issue or the difficult choices that must be made, as illustrated by the COVID-19 pandemic. We introduce a dualist consequentialist approach, weighing utility at both a population and individual level, which may provide a better framework for considering the justification for liberty restrictions. While liberty-restricting measures may be justified on the basis of significant benefits to the population and small costs for overall utility to individuals, the question of whether it is acceptable to discriminate should be considered separately. This is because the consequentialist approach does not adequately account for the value of equality. This value may be protected through the application of an additional proportionality test. An algorithm for making decisions is proposed. Ultimately whether selective liberty-restricting measures are imposed will depend on a range of factors, including how widespread infection is in the community, the level of risk and harm a society is willing to accept, and the efficacy and cost of other mitigation options.

A range of measures exist to mitigate a pandemic. Here, we focus on liberty restrictions aimed at reducing social contacts to prevent particular groups of people from contracting an infectious disease. An example of such an approach is described in a modelling analysis by Ragonnet *et al* conducted in relation to COVID-19, which projects the outcomes of having different restrictions on movements for different age groups.^1^ Its results suggest that an age-selective mixing strategy may make it possible to achieve herd immunity with a much lower level of mortality than without such age-selective measures. However, this theoretical approach can only be implemented by dividing society and severely restricting the liberty of older people. This article explores the ethical challenges that arise from a discriminatory approach such as this.

In relation to COVID-19, the modelling conducted by Ragonnet *et al* suggests that an age-selective strategy may have been more effective in mitigating the disease than the measures applied in some countries.^1^ In the context of widespread vaccine availability and delivery, there is likely to be less to gain from developing a level of population immunity through infection. However, vaccine supply is currently restricted and the vast majority of jurisdictions are not pursuing an elimination strategy—such that the current approach can be considered as either suppression or mitigation. As countries roll-out vaccines, there will be further substantial transmission before vaccination-induced population immunity can form the basis of control. Given this situation, it remains critically important to consider how best to optimally mitigate transmission, even if this forms part of a broader plan that includes vaccination, rather than the whole strategy.

We first explore why liberty-restricting measures might be acceptable during a pandemic and then consider the situations in which discriminatory liberty-restricting measures may be acceptable. Finally, we consider the complexity of decision making in a pandemic and the challenges of weighing risks and benefits to identify when it may be acceptable to implement a discriminatory liberty-restricting measure. We further propose an algorithm to assist decision makers to consider the implications of such policy choices.

## Selective strategies

Selective mixing strategies aim to reduce the morbidity and mortality caused by a virus by limiting the social contacts of those most vulnerable to the disease. Under a selective approach, liberty restrictions are imposed on groups, rather than applying the same restrictions across the population, so that of the infections that do occur, more accrue in lower risk groups.

Ragonnet *et al* suggest that age-selective mixing strategies can have a profound effect to reduce the ultimate mortality cost of the COVID-19 pandemic.^1^ An epidemiological model was used to analyse the epidemics in six European countries that experienced a substantial COVID-19 burden in March and April 2020. The model incorporated age-specific differences in susceptibility to infection and mortality risk. It used previously published age-specific mixing matrices that provide an estimate of the frequency with which different age groups come into contact with one another, along with empirical data on population movement and case and death rates in each of the analysed countries.^2^ An optimisation analysis was then conducted, which sought to determine the age-specific mixing strategy that would result in herd immunity with the lowest mortality effect.

The analysis suggested that drastically reducing the social contacts of people over the age of 50 would result in herd immunity with the lowest mortality costs. To achieve these results, substantial reductions in social contacts would need to be enforced for those over 50, while younger age groups could be permitted to socialise normally. The reduction in social contacts would occur primarily through reducing the number of contacts, but this could be complemented by taking preventive measures to reduce the per-contact risk (eg, mask-wearing, meeting outdoors). Such measures would be similar to the first population-wide lockdown in place in the UK from March 2020 until June 2020, which was estimated to result in a 74% reduction of contacts across the population.^3^

The results of the optimised scenario were compared with an unmitigated scenario, in which populations continued to interact as they did prior to the pandemic. The mortality outcomes between the intervention and the unmitigated scenarios were dramatically different. In the UK, the unmitigated scenario projected around 470 000 deaths over 15 months, compared with 47 000 deaths under the optimised scenario, a 10-fold difference. Importantly, the intervention scenario saw high numbers of infections (as required to develop population immunity), although hospital capacity was maintained at levels similar to what was experienced in March and April in most settings. So, although the infection rate would be large, infections accruing predominantly within younger people would minimise demand on the healthcare system and COVID-19 mortality.

The modelling suggested that if such an approach had been adopted from October 2020, it could have been expected to result in additional COVID-19 deaths after 6 months of age-selective restrictions: approximately 5000 in Belgium, 28 000 in France, 45 000 in Italy, 23 000 in Spain, 3000 in Sweden and 48 000 in the UK. Each of these deaths would mean the premature loss of a person, who has intrinsic value themselves and who would likely leave behind many bereaved friends and family. This is a significant cost, and many may think that a policy that projects these outcomes should not be pursued. However, on the 4 April 2021 the COVID-19 death toll since October in these countries was: 13 153 in Belgium, 64 830 in France, 75 136 in Italy, 43 907 in Spain, 7605 in Sweden, and 84 845 in the UK.^4^ In all countries the death toll is already higher (and continuing to rise) than the projections from the modelled age-selective approach. In reality, the approach that was pursued often consisted of a yoyoing from ‘no restrictions’ to a blanket population-wide lockdown.

The theoretical approach described by the model works through harnessing natural immunity to rapidly and more efficiently attain herd immunity, by allowing more infections among those who have the greatest rates of social contact but are also least susceptible to severe disease. A potential benefit of this would be permitting a faster return to normality with consequent benefits for both health and general societal well-being.^5^ However, there are some important negative effects of allowing infection that are not captured by the model: the morbidity following infection and the increased risk of new variants arising that may make the pandemic harder to control. Some of these new variants are more transmissible and possibly more lethal than the original strain, and there is concern that a variant may develop ‘immune escape’, whereby the immune response after infection or vaccination is insufficient to protect against repeat infection.^6^ These are important considerations. All things being equal, the fewer people who contract COVID-19 the better. But, as is discussed further below, the issue is not merely about identifying the best strategy to avoid risk. Instead, it is necessary to balance the avoidance of risks against the cost of doing so.

Selective approaches may also be relevant if the aim is to reduce infections overall. In the case of COVID-19, the rapid development and roll-out of vaccination in some countries, means there is less to gain from tolerating infection to allow more rapid development of population immunity. Vaccination now offers many countries the opportunity to achieve substantial population immunity without having to rely on natural infection alone. Some countries have pursued an elimination approach with relative success. In Taiwan, Vietnam, New Zealand and Australia, COVID-19 infection rates have remained minimal, with a combination of strict border control and quarantine, frequent testing and rigorous contact tracing. In these settings, minimising COVID-19 infections and applying short-term restrictions to all, regardless of individual COVID-19 risk, to regain elimination in the case of an imported outbreak may be the best pathway while awaiting vaccine roll-out. However, elimination of COVID-19 is unlikely to be feasible if countries are unable or unwilling to implement strict lockdown and border control measures that reduce the risk of imported virus to close to zero. In settings where strict border measures cannot or will not be introduced, eliminating community transmission without ongoing internal movement restrictions seems much less feasible. There are some social interactions that are essential for society to function, even where viral transmission is endemic. In these settings, options that minimise the health burden while acknowledging that some social contacts (and therefore some transmission) are inevitable become more important. In these scenarios there is still a benefit in prioritising preventing infection in people at greater risk of severe disease—especially prior to the development and distribution of vaccines, but even as vaccination programmes are rolled out.

When making decisions during a pandemic, governments are faced with inevitable uncertainty about the future spread of the disease and the possibility of the rapid development of a vaccine. But the yoyoing from ‘no restrictions’ to blanket restrictions approach while waiting for a vaccine to be developed should not be considered the only option. Considering the ethics of selective liberty-restricting measures may help to guide future public health responses. For COVID-19, risk primarily correlates with older age, but in future pandemics risk may correlate with other features, including different age groups, sex or other genetic or environmental factors.

## When are liberty-restricting measures acceptable?

Liberty-restricting measures are a commonly accepted public health tool. Measures such as quarantine have been used for centuries to prevent the spread of disease.^7^ During the COVID-19 pandemic, liberty-restricting measures have not just been used to prevent diseases from entering populations, but also to slow or stop its spread by reducing social contact in settings in which the infection is already established. In the age-selective mixing strategies described above, liberty restrictions are imposed selectively on particular groups of society specifically in order to prevent older and more vulnerable people from contracting COVID-19. This strategy uses liberty restrictions to limit the negative impacts of a disease on a particularly vulnerable group while still accepting some spread.

When considering the acceptability of public health measures, liberty-restricting measures are often justified on the basis they are necessary to prevent harm to others and the least restrictive option available—the so called ‘least restrictive alternative’.^8 9^ In this section, we argue that this does not allow consideration of all the relevant issues and that the acceptability of liberty restrictions should be assessed through a dualist consequentialist approach.

### The harm principle

Mill argued the sole ground for interference in liberty is to prevent harm to others and that harm to self is never a sufficient ground.^10^ This recognises that people should be free to make their own decisions, including to identify and weigh risks to their own health. The challenge of infectious diseases is that people are not just the victims, they are also the vectors, and so their infection poses a risk of harm to others.^11^ This challenge is amplified in a pandemic, as people pose a risk to others through the potential spread of the disease and by contributing to overwhelming the healthcare system if they become ill.^12^ This means that when liberty-restricting measures are imposed during a pandemic they are not merely paternalistic measures to prevent people from failing to protect themselves. During the COVID-19 pandemic, a number of liberty-restricting measures have been justified on the basis that they will limit the spread of the disease and so prevent the health system from being overwhelmed.^13^ On these grounds, various coercive measures could be adopted, including quarantine, isolation, lockdown and surveillance.^14^ Under this framing, the extent to which liberty-restricting measures are justified depends on the level of risk and potential severity of the harm.^14^

But the harm principle does not address what level of liberty restriction is justified for what level of risk of harm. It also does not specify how various harms against different groups should be balanced. As Verweij identifies, this creates the challenge of delineating between reasonable steps and ‘excessive precautions’.^15^ One way of addressing this has been the ‘least restrictive alternative’.^8^ This states that for a given public health goal, we should adopt the measure which least restricts liberty to achieve that goal.^16^ The problem with this is that in many cases, greater liberty restrictions will achieve greater benefits. So which policy is justified?

### A dualist consequentialist approach

Under the harm principle a person risks causing harm to others by infecting them with a disease or by using health resources if they become ill, and liberty restrictions are justified to prevent this harm if the risk of morbidity and mortality mean the harm is sufficiently serious.^14^ There are two problems with this harm-based approach. The first problem is that it suggests everyone faces the same risk if they contract the disease and that they pose the same risk to others. As the modelling demonstrates, the issue is actually more complicated than identifying when the disease burden is so high that it justifies liberty-restricting measures to avoid the spread of the disease.^1^ This is because the disease burden may vary depending on who contracts a disease.

The age-selective mixing strategy explored by the modelling does not aim to reduce the spread of the disease generally, but to prevent particular people from contracting the disease in order to reduce the disease burden. Similarly, an age-selective approach that aims to reduce infections overall, but to achieve this to a greater extent among older people, aims to shift infections from the older to the younger population, and so is also concerned with preventing particular people contracting disease. This reflects a consequentialist concern with reducing the overall negative impacts of the disease. Under this consequentialist perspective, the liberty restrictions in an age-based mitigation strategy are justified on the basis they may result in a 10-fold difference in mortality compared with if there were no liberty restrictions. This consequentialist perspective has one advantage, that it provides a basis to consider the variable impacts of the disease on different people.

A focus on preventing harm to others suggests the management of a pandemic should consider preventing people from acting as vectors. Quarantine measures reflect this approach and focus on preventing people spreading a disease in a vulnerable population that has not yet been exposed to the infection. But rather than focusing on the vector, a consequentialist approach recognises it is possible to limit the harm caused by the disease by focusing on the victim. An individual has limited control over whom they infect, but public health measures may limit the extent to which those most at risk are exposed to the disease.

The second problem is that it does not provide any way of balancing liberty restriction against the harm prevented. The least restrictive alternative is one way of specifying the level of liberty restriction. But in practice, greater levels of liberty restriction will yield more utility.^17^ Another way of addressing this problem is to add a proportionality requirement: liberty restrictions must be proportionate to harm averted.^18^ But again, no principled way is provided of deciding proportionality.

A consequentialist approach is preferable to the harm principle because it enables a balancing exercise at a population level that aligns more closely with the aims of public health. Public health aims to protect and promote the health of the population.^14^ Public health measures are not simply aimed at ensuring people do not harm others and achieving this in the least restrictive manner. An ethical framework is needed that defines the circumstances in which it would be appropriate to pursue population-wide benefits in light of the costs of doing so. This may be achieved by first considering the utility of a measure at the population level and then considering the costs to relevant individuals of achieving these benefits. These two steps are described below. For the purposes of this framework, the population is understood as the aggregate of individuals within a particular jurisdiction and an individual is a person to whom a measure would apply. It is necessary to assess the net utility of a measure across the population and the cost to individuals separately because although the individual may be part of the population, imposing measures on particular individuals may result in disproportionate costs to those individuals.

The famous British philosopher Henry Sidgwick described the Dualism of Practical Reason. Sidgwick identified that there are two reasons for action: morality and self-interest.^19^ Morality has to do with well-being of the group or all members of society (impartially considered); self-interest is related to an individual’s own well-being (prudence). As another British philosopher Derek Parfit notes, ‘[w]hen reasons of these two kinds conflict, neither could be stronger. We would always have sufficient or undefeated reasons to do either what would be impartially best or what would be best for ourselves’.^20^ We will not address this ‘profoundest problem’ of resolving this conflict—rather we adopt a consequentialist approach that has both good overall consequences for a group and for individuals in that group. We call this a dualist consequentialist approach. (Note, we are silent on whether it is a maximising consequentialist approach.)

### Population-level utility assessment

Rather than simply assessing whether there is a sufficient risk of harm to warrant liberty-restricting measures, it is necessary to consider the utility of a measure and whether the net utility is greater than other available options. Three factors are relevant to this assessment:

- The gravity of the threat to public interest.
- The expected utility of the measure compared with other measures.
- The extent to which the expected utility outweighs the restriction of liberty.

At a population level, a measure will be justified if the comparative expected utility justifies the restriction of liberty. For example, requiring everyone to wear masks might be a reasonable restriction for everyone, even if some are unlikely to become ill or pass on the virus. Such an intervention has the potential to reduce disease burden and is a small liberty restriction. This weighing exercise recognises that it is not appropriate to focus solely on the number of lives saved or implementing the least restrictive measure, but rather that a balance must be struck.

### Individual costs

Utility at a population level cannot always be given priority.^21^ A key objection to a utilitarian approach is the risk that it will result in utilitarian calculations in which people’s liberty and well-being will be restricted whenever this would result in a net overall benefit to society.^14^ Utilitarianism is strictly impartial and only concerned with the moral perspective. This may mean that particular groups of individuals are forced to make significant sacrifices in order to achieve marginal social gains or that the burdens of achieving public health aims may continually fall on the same group. This would be unfair. This issue may be overcome by considering the outcomes at an individual level and the overall cost of liberty restrictions to an individual compared with the benefits to others.

Giubilini *et al* argue that ‘If the cost (including foreseeable risk of significant disability or death) to someone of performing an action X (or of refraining from performing an action Y) is sufficiently small to be reasonably bearable, and the resulting benefit to other people (or harm that is prevented) is large relative to the cost, then the agent ought to do X (or not do Y)’.^22^ This may be described as a case of ‘easy rescue’ and they suggest that if a person has a moral obligation to do something, this provides a stronger basis for state intervention to compel the person to do it.^22^

For example, the modelling suggests that restricting the liberty of those over the age of 50 may save over 400 000 lives in the UK compared with the unmitigated scenario.^1^ For an individual over the age of 50, this benefit is achieved at the cost of their liberty. But the liberty restriction also benefits them by preventing their exposure to a disease that poses a particular risk to them. Importantly, this age group is also the most vulnerable to COVID-19 and so the benefit to them is significant. Arguably, it is a net overall benefit to them. At an individual level, while the cost of the liberty restriction may be great, this must be weighed against the personal benefit of avoiding the disease. There may even still be a net cost to the individual, but this may be outweighed by the benefit to others and reasonable if the net cost is small. For people over the age of 50, this may be a net benefit or at least a case of ‘easy rescue’, in which the overall cost to the individual of saving others is relatively small, compared with the benefit to others.^23^

At an individual level, the weighing of costs and benefits for those under the age of 50 is different. This is because imposing liberty restrictions on people under the age of 50 will not directly benefit them in the same way because COVID-19 does not pose the same risk to them. Despite this reduced personal benefit, those under the age of 50 would still incur the same costs from liberty restrictions as well as costs to their well-being in other ways. For example, the closure of schools during the COVID-19 pandemic will significantly harm children and may substantially impact their development.^24^ As there are less benefits for someone under the age of 50 in being isolated, the relative cost of liberty restrictions may outweigh the potential benefits to others. For those under 50, liberty restrictions may be a more difficult rescue.

Considering the costs and benefits of a measure at both a population and individual level ensures that individuals are not forced to bear disproportionately high costs to achieve marginal social gains and ideally benefit from them. A dualist consequentialist perspective supports age-selective liberty restrictions in the COVID-19 pandemic. There are of course challenges in identifying relevant costs and benefits and making generalisations across the community about the value of different costs and benefits to individuals.^25^ For example, the cost of liberty restrictions may vary significantly across individuals, including among people of the same age. But this challenge also arises under the application of the harm principle and least restrictive model. In the assessment of the impact of any policy across a population it is necessary to make generalisations.

### Dualism about value

One objection is that liberty restriction of those under 50 nonetheless benefits most people because COVID-19 still represents a lethal risk. This objection ignores other non-COVID-19-related costs to a person’s well-being from liberty restrictions. Even if it was accepted that there was an overall benefit in terms of well-being to those under the age of 50 in having their liberty restricted, there is a second way in which selective lockdown can be justified. Well-being is plausibly not the only value. Liberty or freedom is also a value. Dualism about value would consider two relevant values to be promoted or maximised: well-being and liberty.

If we accept not only dualist consequentialism but Dualism about Value, selective liberty restriction can be justified. For those over 50, the large increment in well-being outweighs the modest loss of liberty. Lockdown is of overall value (utility) for them. For those under 50, the small increase in well-being (or expected well-being, more precisely) is offset by the modest restriction of liberty. Lockdown has net negative expected overall utility.

In conclusion, if we consider both the individual and the group, and consider both liberty and well-being as of value, then selective restriction of liberty of those over the age of 50 can be ethically justified in the pandemic.

## Are discriminatory liberty-restricting measures acceptable?

### The value of equality

If a dualist consequentialist approach is accepted, liberty restrictions may be imposed to reduce the disease burden. The modelling demonstrates that the impact of restrictions may vary depending on the characteristics of those whose liberty is restricted.^1^ This leaves the question of whether it would be acceptable to restrict the liberty of a group of people on the basis of particular characteristics in order to reduce the disease burden? The extent to which discrimination is acceptable is identified as a separate consideration because, as Childress *et al* identify, moral concerns that justify public health goals, such as producing benefits and preventing harms may conflict with other moral concerns, such as equality.^26^ Equality has value because it demonstrates respect for a person as a distinct individual. Hellman argues discrimination is wrong when it demeans the person affected.^27^ A person is demeaned when they are not treated with the same concern and respect.

While consequentialists such as utilitarians do value equality as equal consideration of interests (as we do here), selective restriction of liberty may appear to violate Aristotle’s principle of equality to treat like cases alike, unless there is a morally relevant difference. Failing to treat like cases alike constitutes unjust discrimination.

Upholding equality may mean that even if a measure optimally balances the benefits and harms at a population level and an individual level, it may still not be appropriate to implement the measure because it involves unjust discrimination. Childress *et al* propose five ‘justificatory conditions’ to proceed with public health measures in these circumstances: effectiveness, proportionality, necessity, least infringement and public justification.^26^ These conditions identify relevant considerations. We now provide a test to assess when it may be appropriate to discriminate. While balancing benefits and harms may ensure that each individual’s interests are considered equally, the test proposed ensures that particular groups of people are only treated differently when this is justifiable.

### Relevant differences

Some people are more likely to become severely unwell and to die. If the aim of a measure is to reduce disease burden, it may be acceptable to differentiate between people based on their risk of morbidity and mortality. In the case of COVID-19, there is a clear correlation between age and risk of death. The risk of the 20–24 years old dying as a result of an episode of infection with SARS-CoV-2 has been estimated to be 4/100 000 or 0.004%;^28^ lower than the yearly risk of dying in a car accident in the USA.^29^ Considered in isolation, this risk does not warrant liberty-restricting measures. But the risk of a person over the age of 85 dying is estimated at 7%.^29^ This is a significant risk of death that may warrant liberty-restricting measures. What the age-based mitigation strategy highlights is that it is not necessary to impose the same restrictions on the whole population in order to avoid this risk. Instead, measures may focus on preventing those most at risk from contracting the virus. This would mean restricting the liberty of those who face a 7% risk of death, but not those who face a 0.004% risk.

An objection to a consequentialist approach that reduces the negative impacts of a pandemic by focusing on who contracts the disease is that this fails to respect the individuality of those in a higher risk group. But restricting everyone’s liberty to avoid the same risk is levelling down equality.^30^ People at significant risk of death from a pandemic disease are likely to need to submit to liberty restrictions in order to avoid this risk, but this does not necessarily mean everyone should be subjected to the same liberty restrictions. It is common for governments to impose liberty-restricting paternalistic measures on particularly vulnerable groups in order to protect them and others. But implementing such measures every time a particular group was identified as posing a risk to others would significantly undermine equality. The issue is when this discrimination is justified.

### Two kinds of harm

As we have pointed out, the annual risk of dying in a car accident for someone under 30 is about the same as dying of an episode of COVID-19.^31^ Now the risk of other groups dying in car accidents is much higher: those who are drunk, abusing drugs, have epilepsy or other underlying medical conditions, and the elderly. However, the response is not to ban all driving of cars because some groups have a higher chance of dying or being injured in a car accident. The response is to ban those at higher risk.

There is one obvious disanalogy between driving and COVID-19. In the case of driving, those at higher risk are both a higher risk to themselves and to others. This is not the case for most of the lower risk age groups in relation to COVID-19—they are not at a higher risk of harming themselves, but do put others at higher risk. On this basis, everyone’s liberty should be restricted because everyone is at an elevated risk of harming others.

This is an important point, but there is a relevant response. In both COVID-19 and driving, there are two kinds of harm that may be caused. The first is the direct harm: colliding in a car or passing on the virus. The second is using limited health resources for hospital care following a not immediately fatal incident. Now generally, we allow people to take risks that expose them to utilising health resources. Healthcare is there to facilitate people realising their plans of the good life. But unlike driving, a pandemic is an extreme emergency. In such a situation, the state is entitled to restrict freedom to prevent overwhelming of the health system in order to ensure people can continue to access healthcare. This is not the case in ordinary driving where no extreme emergency exists.

So while we don’t normally take ‘use of limited health resources’ to be a decisive factor in restricting liberty, in a pandemic we assert that it can be. And it is on this ground that those who are more at risk (over 50) present a kind of harm that others who are under 50 do not present (even if both present the same risk of spreading the virus). And it is on this ground their liberty can be restricted just as in the driving case.

Nonetheless, some will still object that restricting the liberty of a group is unjustified discrimination. Although the different risks posed to different age groups may be stark, this does not necessarily mean it is ethically acceptable to discriminate between these groups or to impose harsh liberty restrictions on those over the age of 50. It is this question we will now address.

### Historically wrong discrimination

In discussions about quarantine and other coercive measures to limit the spread of an infectious disease, concern about past practices has encouraged a focus on equality. Fairchild *et al* explain ‘One way to understand the past approach to disease and containment is to read it in a story of blame and social division’.^32^ For example, the suspicion of a plague-related death in Chinatown in San Francisco in 1900 led to the evacuation of white residents while Chinese residents were blockaded within the district.^32^ Such treatment is plainly wrong. People were treated differently on the basis of their ethnicity even though this had no effect on the spread of the disease.

These historical wrongs have justifiably encouraged a focus on equality. For example, Selgelid argues that liberty-restricting measures must be used in an equitable manner.^14^ Selgelid suggests this may be achieved by avoiding applying such measures in a discriminatory manner against marginalised groups or by requiring that such measures are only used in a discriminatory manner when there is strong justification. This would recognise that some members of society require special protection.^14^ Viens *et al* argue that if liberty-restricting measures are employed in a discriminatory fashion this will violate a state’s obligation not to discriminate and so is unjustifiable.^8^ Viens *et al* suggest this is the case ‘even if restrictive measures are implemented in a way that makes them measurably successful overall in containing the contagion’.^8^

These broad statements appear to overstate the extent to which it is wrong to implement measures that treat people differently. Discrimination on the basis of a particular characteristic may be morally permissible when that characteristic correlates with a morally relevant difference. For example, during the COVID-19 pandemic, people living in the Australian state of Victoria have faced significantly harsher restrictions on their movement than the rest of Australia.^33^ This is not unjust discrimination against Victorians, but rather reflects the fact that the virus was more prevalent in Victoria, such that it was appropriate to differentiate on the basis of geographical location.

Differential treatment on the basis of relevant differences is not just permissible, but is necessary to achieve equitable outcomes. As Fairchild *et al* identify, universally applied social distancing measures during COVID-19 created a superficial equality, but the impact of the measures were ‘profoundly unequal’.^32^ Restrictions on when it is acceptable to leave home have a different impact on someone who has a stable home environment and has the option to work from home, compared with someone who has neither. Just as there are relevant differences in assessing the impact of liberty restrictions on particular groups, there are also relevant differences in assessing the risks of particular groups contracting the disease.

### Proportionality

The identification of higher risk groups is not necessarily a sufficient basis to discriminate. It will always be possible to identify a particular group that is at higher risk from an infectious disease and accepting liberty restrictions in each of these cases would significantly undermine equality. The issue is identifying when the difference is significant enough to warrant discrimination. This may be understood in terms of proportionality. When would discrimination be a proportionate response?

Human rights law recognises that a human right may be limited when this would be proportionate. The tests developed under human rights law to determine whether the limitation of a right is proportionate may provide practical guidance in determining when a discriminatory measure is appropriate. Human rights instruments such as the European Convention on Human Rights recognise the right to equal enjoyment of human rights, including the right to freedom of movement, and so are relevant to discriminatory liberty-restricting measures.^34^ There are a number of variations of the proportionality test, but the four limb test developed in the UK is discussed here.^35^ This is not explored from a legal perspective, but is presented as a well-developed framework for considering when discrimination may be proportionate. This suggests that a measure will be proportionate if:

1. The objective was sufficiently important to justify limiting a fundamental right.
2. The measure designed to meet the objective was rationally connected to it.
3. The means used to impair the right or freedom were no more than is necessary to accomplish the objective.
4. The measure strikes a fair balance between the rights of the individual and the interests of the community.

If these tests are applied to the historical instances of discrimination discussed above, it is clear they would fail on the second and third limb. This is because there was no rational connection between ethnicity and those diseases and because it was no more necessary to restrict the liberty of these groups than others. However, this does not mean it will never be proportionate to discriminate.

The measures proposed in the age-selective mixing strategy may be proportionate under this test. Limiting the number of deaths that occur during a pandemic may be sufficiently important to justify limiting the right to equality and so may fulfil the first limb. Under the second limb the modelling demonstrates there may be a rational connection between restricting the liberty of one group of people and limiting the negative impacts of a pandemic.

The application of the third limb raises a number of issues. If the objective is to limit morbidity and mortality, an age-selective mixing strategy is not strictly necessary as there are a range of other options, such as non-selective strategies. But each of these options also have negative effects and conflict with other moral concerns. It may be argued that if the objective is to limit morbidity and mortality, it is necessary to prevent people over the age of 50 from contracting the virus because of the greater risk the virus poses to them. Until those most at-risk are protected with an effective vaccine, some form of liberty restrictions are necessary.

The fourth limb is the broadest and may create some ambiguity, as the relevant ‘interests of the community’ are undefined. In relation to the age based mitigation strategy, this would achieve substantial benefits in reducing morbidity and mortality and preventing the health system from being overwhelmed.

The proportionality test may provide guidance about the acceptability of future discriminatory liberty-restricting measures. The measure may be acceptable if:

1. The objective is to limit the disease burden.
2. The measure is designed to prevent those who are most at risk from contracting the disease.
3. The liberty restrictions imposed must be no more than are necessary to limit exposure to the virus.
4. Liberty-restricting measures on high-risk groups would significantly reduce the utilisation of limited health resources and the mortality rate of the disease, which would otherwise result in a large number of deaths.

This test recognises the value of equality and that the issue should not be reduced to a question of health benefits versus liberty restrictions. It is important that each person is respected as an individual and that they are not arbitrarily discriminated against.

### Broader justice considerations

Gostin and Berkman argue ‘in the exercise of compulsory powers, distributive justice requires a fair allocation so as not to burden unduly particularly vulnerable populations’.^36^ It is necessary to account for the extent to which measures may exacerbate existing inequalities. For example, in Australia in order to mitigate the spread of COVID-19, the residents of densely populated community housing were ordered to remain in their homes for over a week.^37^ Based on their geographical location, people living in the community housing were at greater risk of having COVID-19 and of spreading it. But this risk arose from pre-existing inequality and the decision to effectively place people under house arrest placed an additional burden on this group of people because of this.^37^ What appears unjust about this scenario is that social conditions resulting in inequality were what placed the people in the community housing at higher risk.

In relation to an age-based mitigation strategy, older people are already more likely to be socially marginalised and restricting their liberty is likely to further contribute to this. This must be a relevant consideration, but it may also be differentiated from the case of community housing. This is because people over the age of 50 are at greater risk from COVID-19 for reasons unrelated to social inequalities. To address concerns about distributive justice, an additional limb may be added to the proportionality test, requiring the reason the group faces a higher risk from the disease should not be the direct result of social inequality.

While this distinction may appear promising in distinguishing between acceptable and unacceptable discrimination, the issue of risk factors arising indirectly from social disadvantage remains. For example, obesity often places people at higher risk from particular diseases, but there is also often a correlation between obesity and social disadvantage.^38^ Would imposing liberty restrictions on people on the basis of body-mass index also be unjust if there was a much greater risk to people who were obese? Although this is a valid concern, it may not be possible to avoid discrimination on the basis of risk factors arising indirectly from social disadvantage unless every measure is applied universally. But, as discussed above, this will also have an unequal impact and is likely to also result in further disadvantage. Instead, in instances in which risk factors arise indirectly from social disadvantage efforts should be made: (1) to mitigate the extent to which a group is placed at a further disadvantage through any liberty-restricting measures and to address this social disadvantage; (2) where this is not possible, ensure that liberty-restricting measures are not selectively applied to the disadvantaged group, further increasing overall disadvantage (as happened in the community housing example cited above).

### An algorithm for decision making

**Figure 1:**
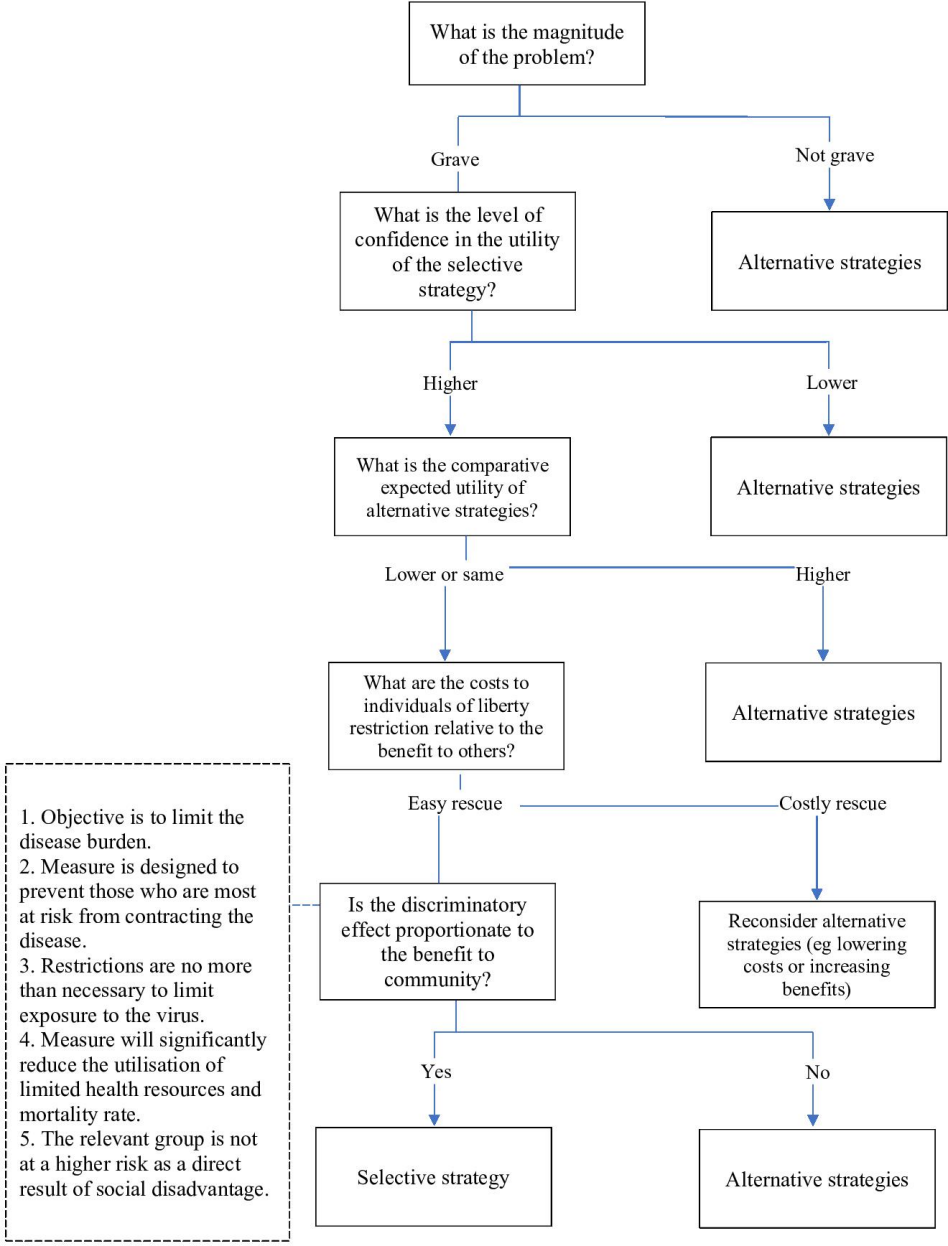
Algorithm for liberty-restricting public health intervent.

Below is an algorithm (figure 1) which captures these considerations for determining when liberty-restricting measures may be acceptable. This recognises liberty-restricting measures should only be implemented when the threat posed is sufficiently grave, that the costs and benefits must be weighed at the community and individual level, and that discriminatory measures should only be imposed if they would be a proportionate response.

## When is it appropriate to implement discriminatory liberty-restricting measures?

The algorithm provides a framework for making decisions about when discriminatory liberty-restricting measures may be acceptable. But the question of when such measures should actually be implemented is more complicated. Weighing the relevant risks and benefits to the community may depend on a range of factors, including the alternative options available. In future pandemics, there may also be more effective contact tracing or rapid specific and sensitive viral detection that may reduce the need for liberty restrictions.^39^ We now discuss some of the additional factors relevant to a government’s assessment.

### Vaccine availability

Achieving herd immunity through a safe vaccination programme is ideal. However, as the case of COVID-19 demonstrates, vaccines take time to develop and vaccinating the population to achieve herd immunity also takes time. While some countries have vaccinated their population relatively rapidly, these are exceptions.^40^ Vaccine manufacturing capacity is limited, and estimates in April 2021 suggest that there will not be enough vaccine for all people globally until 2023 or 2024.^41^ During this time, there will inevitably be further transmission and natural immunity will contribute substantially to achieving herd immunity. An effective vaccine is only an additional tool to mitigate a pandemic and consideration must be given to how it should be most effectively utilised. This means that age-selective mixing strategies may still be appropriate while a vaccine is being developed or rolled out. Van Bunnik *et al* provide an example of how vulnerable populations may be shielded while liberty restrictions are slowly eased.^42^ Such a strategy could be used in conjunction with vaccination. Whether differential treatment based on age, or any other characteristic, is appropriate may depend on a range of factors, including the risk of harm posed by the vaccine, the likely effectiveness of the vaccine (especially relative to natural immunity), the uptake of the vaccine, the relative protection offered by vaccine, and the availability of the vaccine.

### Acceptable risk and harm

The choice facing governments in a pandemic is not simply whether to impose liberty restrictions, but also to decide what level of mitigation is appropriate. The measures adopted will depend on the level of risk and the level of harm a society is willing to accept. Governments could implement measures to attempt to avoid all infectious disease related deaths. But measures such as liberty restrictions have costs and governments must identify which harms are acceptable given the cost (both economic and social) of mitigating them. Influenza is an example of a disease that poses significant risks to health, having caused 26 408 deaths in the UK in 2017/2018.^43^ But the UK does not consider preventing these deaths warrants liberty restrictions, or at least that the cost of liberty restrictions is too great to prevent these deaths. By contrast, the modelling suggests without mitigation strategies COVID-19 may cause around 470 000 deaths in a 15-month period.^1^ Clearly this substantially higher death rate may justify more substantive measures. As the modelling demonstrates though, the choice is not just between accepting 470 000 deaths and zero deaths. Instead, different measures are likely to reduce the death rate in different ways, but there will also be different costs to these measures. The challenge for the government is identifying the point at which a measure achieves an acceptable risk at an acceptable cost.

The challenge of making this decision is evident in the example of the choices that were available to manage COVID-19. In countries that have been unable to eliminate COVID-19, imposing strict and prolonged liberty-restricting measures on the entire population may have resulted in the greatest number of lives saved, but also has the greatest cost in terms of the number of people whose liberty is restricted, and how severely. The age-selective mixing strategy that restricts the liberty of those over the age of 50 would have resulted in fewer lives being saved, but also would have come at a smaller cost in terms of liberty restrictions and sacrificed well-being of those under 50. Given that those over the age of 65 are at an even greater risk than those aged over 50, an age-selective mixing strategy that restricts the liberty of those over the age of 65 may also have been considered. This would have resulted in even fewer lives being saved, but also fewer people would have faced liberty restrictions. Which option is preferable depends on what a government identifies as an acceptable number of deaths at an acceptable cost.

### Exposing particular groups to risks

A measure focused on reducing the burden of the disease carries a range of different risks to measures that focus on reducing the spread of the disease. For example, the age-selective mixing strategy would pose distinct risks. Under the strategy, COVID-19 would be allowed to spread through the under 50 year-old community. This may increase the risk to older people because of the higher prevalence of the disease in the community. As Hughes identifies, this means that the benefits derived from placing liberty restrictions on everyone may be greater than the benefits derived from an age-selective mixing strategy.^44^ The age-selective mixing strategy also exposes people under the age of 50 to the disease on the basis that the disease poses a smaller risk to them, although this risk may not be negligible for all those aged under 50. In order to assess the acceptability of a particular discriminatory liberty-restricting measure, it is necessary to assess the additional risks posed by the measure and whether these are more serious than the risks posed by alternative measures.

The mere fact that a group may be exposed to a potentially avoidable risk to achieve a greater good should not prevent the adoption of a measure. For example, young people are currently encouraged to receive influenza vaccination in order to limit the spread of influenza across the population even though this vaccination poses a risk to them. Exposing young people to this risk is considered acceptable in order to achieve the greater good of preventing those more susceptible to severe illness from contracting influenza. This is because both the risk is sufficiently small and there is some benefit to young people of being immune to influenza.^23^ A dualist consequentialist approach supports vaccinating young people against influenza to benefit older people. Again, this reflects the need to balance risks and the broader costs of measures to mitigate the impact of disease.

### Protecting the health system

A significant concern in a pandemic is the capacity of the healthcare system to continue to treat all those who require treatment. The system may be overwhelmed by the number of infected people, but also because as the disease spreads health practitioners are placed at higher risk and if they become unwell the capacity of the healthcare system may be reduced. Measures that allow infection in the population may place the health system at greater risk of being overwhelmed than measures focused on limiting the spread of the disease in the community.

### The challenge of uncertainty

During a pandemic, decisions must be made when there is considerable uncertainty about the risk posed by the disease, when a vaccine may be developed, and the future progression of the disease. Providing more certainty in the progression of the pandemic by achieving herd immunity may bring value. However, the uncertainty cuts both ways. Herd immunity achieved by exposing people under the age of 50 to a virus when the long-term health effects of the virus remain unclear also presents risks. The risk posed by development of new variants is another area of uncertainty, as is the duration of immune protection. In making decisions, policy-makers are ultimately left to determine which risks they are willing to accept on behalf of society.

## Conclusion

In this paper, we have not sought to argue that selective mixing strategies should be employed. We have created a dualist consequentialist framework where selective restriction of liberty could be justified. Whether it is, or not, will depend on the relevant empirical facts. There are no doubt other arguments (some based on intergenerational desert and justice arguments) which would support age-selective liberty restrictions, but in this paper we have concentrated on when consequentialist considerations could justify selective restriction of personal liberty.

In order to identify appropriate responses to a pandemic, governments should adopt a consequentialist approach with the aim of reducing the disease burden to an acceptable level of harm. What constitutes an acceptable level of harm will depend on a range of factors, including the morbidity/mortality impact of an unmitigated epidemic, the extent to which this harm could be reduced with selective measures, the extent to which the disease has spread already in a population, the political and geographical features that impact the ability to eliminate and prevent re-introduction of the virus, the harms of countermeasures, and the resources available to the government. Selective restriction of liberty is justified when the problem is grave, the expected utility of the liberty restriction is high and significantly greater than the alternatives and the costs of the liberty restriction are relatively small both at both a population and individual level. That is, when the need for liberty restriction is considered an ‘easy rescue’. Discrimination can be justified under these conditions when it is proportionate and limited to a very specific public health challenge.

## Data Availability

There are no data in this work.
